# High Frequency Data Acquisition System for Modelling the Impact of Visitors on the Thermo-Hygrometric Conditions of Archaeological Sites: A Casa di Diana (Ostia Antica, Italy) Case Study

**DOI:** 10.3390/s18020348

**Published:** 2018-01-25

**Authors:** Paloma Merello, Fernando-Juan García-Diego, Pedro Beltrán, Claudia Scatigno

**Affiliations:** 1 Department of Accounting, University of Valencia, Av. de los Naranjos s/n, 46071 Valencia, Spain; paloma.merello@uv.es; 2 Department of Applied Physics, Universitat Politècnica de València, Av. de los Naranjos s/n, 46022 Valencia, Spain; pbeltran@fis.upv.es; 3 Nanoscience & Nanotechnology & Innovative Instrumentation (NAST Centre), University of Rome “Tor Vergata”, Via della Ricerca Scientifica 1, 00133 Rome, Italy; claudia.scatigno@uniroma1.it

**Keywords:** visitors, temperature, relative humidity, thermo-hygrometric balance, continuous monitoring, preventive conservation

## Abstract

The characterization of the microclimatic conditions is fundamental for the preventive conservation of archaeological sites. In this context, the identification of the factors that influence the thermo-hygrometric equilibrium is key to determine the causes of cultural heritage deterioration. In this work, a characterization of the thermo-hygrometric conditions of Casa di Diana (Ostia Antica, Italy) is carried out analyzing the data of temperature and relative humidity recorded by a system of sensors with high monitoring frequency. Sensors are installed in parallel, calibrated and synchronized with a microcontroller. A data set of 793,620 data, arranged in a matrix with 66,135 rows and 12 columns, was used. Furthermore, the influence of human impact (visitors) is evaluated through a multiple linear regression model and a logistic regression model. The visitors do not affect the environmental humidity as it is very high and constant all the year. The results show a significant influence of the visitors in the upset of the thermal balance. When a tourist guide takes place, the probability that the hourly temperature variation reaches values higher than its monthly average is 10.64 times higher than it remains equal or less to its monthly average. The analysis of the regression residuals shows the influence of outdoor climatic variables in the thermal balance, such as solar radiation or ventilation.

## 1. Introduction

For the last decades, preventive conservation of cultural and archaeological sites has been understood as the whole control process of the deterioration factors in order to prevent damage to the cultural heritage (CH) before it occurs and minimize future interventions [1]. It is acknowledged as important for safeguarding CH, both in terms of preservation and reducing the costs of future conservation measures [2]. 

The deterioration process is determined by factors such as the petrographic and chemical characteristics of the materials, the presence of mineral salts and organic substances on the surfaces, air pollution, sunlight, temperature, water content of the surface, etc. [3]. Therefore, preventive conservation requires knowledge of the specific characteristics of a specific CH site (materials, mineralogical conditions, natural ventilation, tourist campaigns, etc.), as well as the set of parameters connected to it, including microclimatic conditions (temperature, humidity, wind, air conditioning, etc.) [4], among others.

As thermo-hygrometric parameters affect chemical reactions, they appear as one of the most influential factors of the conservation of a CH site [5]. Microclimatic monitoring studies have been conducted in churches [6,7,8] museums [9,10,11,12] and archaeological sites [13,14,15,16].

CH has survived for many centuries in conditions that must be considered risky but stable (equilibrium), without avoiding damage to the materials, but resulting from a long adaptation process. Thus, abrupt changes of microclimate parameters interrupting this equilibrium conditions induce further damage to material until a new equilibrium is reached [17].

Therefore, it is important to characterize an archaeological site prior to carrying out comparative studies in the future for preventive conservation, either by regular studies to verify whether the conditions are constant or occasional ones, when the boundary conditions are altered. 

In this vein, the presence of visitors could cause large deviations from the usual conditions [18] as humans can alter with their presence or behavior the aforementioned parameters, affecting the conservation of sites with interest in preservation. In addition, many of these variables are normally controlled for the comfort of visitors, starting then an interesting debate between the prevalence of preventive conservation and human comfort.

A big problem arises when the microclimate has been planned only for the wellbeing of visitors disregarding the needs of conservation that requires a constant climate [18]. Hopefully this has improved in recent decades and the conservation of heritage sites has been prioritized. Therefore, the management and planning for preserving historical buildings is strictly connected to their use, mainly for tourism. 

There are many works that study the impact of visitors in tourist sites with protection interests such as Geoparks and other geological sites [19,20,21,22], museums [18] and other cultural and archaeological sites [16,17,23,24].

Casa di Diana (Ostia Antica, Italy) is a complex building, comprised of several rooms, including a *Mithraeum* (a place dedicated to the cult of the Persian god Mithra during Roman times). Its microclimate behaves like a *hypogeum* (an underground room simulating a cave, used for mithraic rites—as initiations), despite being structurally comparable to a semi-confined environment and being defined as such [15,16]. Casa di Diana is accessible to guided tours with advance booking. 

In the case of hypogea environments, characterized by a great stability (constant humidity and temperature) [25] and the abundance of nutrients providing a suitable niche for phototrophic microorganisms when combined with artificial illumination [15,24] visitors can be considered as a possible factor breaking that equilibrium state.

The results of [24] in the *hypogeum* of San Callixtus Catacombs (Rome) indicate that a sharp temperature increase was due to the automatic ignition of lamps when people passed along the corridors, as well as other effects on air temperature due to human body heat and in CO_2_ due to breathing.

A monitoring campaign of indoor CO_2_ concentration was conducted in Casa di Diana with the aim of evaluate its effects on biological colonization [26,27], determining that the growth of vegetation is concentrated in the lowest area of the *podiae* (seats for the Romans that assisted the sacrificial ritual of killing the bull), which also corresponds to the path followed by visitors.

The CH is conserved in order to be disclosed, contemplated and admired by the visitors being these who maintain it but, nevertheless, they can contribute to its deterioration. It is necessary to reach a consensus of disclosure of our CH in a sustainable manner. This paper deals with a mathematical modeling in order to quantify the influence that visitors can produce in our CH. Considering the thermo-hygrometric conditions of Casa de Diana as a case study. 

A monitoring system, able to record and store large amounts of data (monitoring frequency of 1 data point per minute) was installed [11,28]. The system was comprised of 29 probes, including temperature and relative humidity sensors installed in parallel, calibrated and synchronized with a microcontroller.

As far as we know, this is the first time that a quantitative study is carried out with the aim of modelling the influence of visitors on the upset of the thermo-hygrometric equilibrium of an archaeological site. The methodology proposed in this paper may serve as a reference for other CH sites.

The paper is organized as follows: after the Introduction, a description of the *Mithraeum* of Casa di Diana, the monitoring system, a description of the sample and the analysis procedure are presented in Section 2. Section 3 deals with the mathematical modelling of the impact of the visitors in the upset of the thermal balance. Finally, the Conclusions section summarizes the main remarks.

## 2. Materials and Methods

### 2.1. The Archaeological Site: “Casa di Diana” Mithraeum

The Casa di Diana Region I, Insula III, is a Roman building (130 CE) part of the famous archaeological site of Ostia Antica, located at 23 km from the center of Rome. The building, comprised of *tabernae* (a single room shop covered by a barrel vault within great indoor markets of ancient Rome; normally with overhead lighting openings) and *cenacula* (dining rooms), presents a very characteristic microclimate, especially inside two intercommunicating rooms (the *Mithraeum* and *pre-Mithraeum*). The principal building materials are bricks and pozzolanic mortar aligned with the *opus caementicium* (Roman concrete, based on a hydraulic-setting cement, was a material used in construction during the late Roman Republic until the fading of the Roman Empire) technique [25,26]. The two rooms are characterized by different heights due to the presence at the sides of *podium* [26]. 

The ventilation is natural and comes from several openings. No mechanical ventilation systems are installed. The rooms are covered by a roof and protected against the rain, but small areas are directly exposed to sunlight and outer air due to the openings [16].

The Casa di Diana is visited, one or two days a week, normally at 10:30 a.m., with a mean duration of about 30 min (only 10 min in the *Mithraeum*’s rooms). 

### 2.2. Monitoring System

The sensors consist of probes that contain an 8-pin small-outline integrated circuit (SOIC), model DS2438 [29] (Maxim Integrated Products, Inc., Sunnyvale, CA, USA) that incorporates a temperature (T) sensor with an accuracy of ±2 °C as well as an analogue-to-digital voltage (A/D) converter which measures the output voltage of a relative humidity (RH) sensor (HIH-4000 [30], Honeywell International, Inc., Minneapolis, MN, USA). The sensor has repeatability between measures allowing to detect small variations [11,14]. Three electric wires come out from each probe: one wire for +5 V DC power supply, one for ground and another for data transfer.

Because each DS2438 [29] has a unique silicon serial number, multiple DS2438s [29] can exist on the same data bus. This allows multiple sensors that can be used in the system simultaneously with only one data line (1-wire communication protocol). The sensors, with this communication protocol, are installed in parallel between the data line and ground, allowing a simple and robust wiring.

The HIH-4000 [30] RH sensors, with an accuracy of ±3.5%, were calibrated in the laboratory with a saturated solution of salt [11,14]. The voltage output of the HIH-4000 [30] sensors is measured by one of the four analogs to digital A/D converters of the DS2438 [29]. As specified by the manufacturer, the voltage output is proportional to voltage supply. The exact value of the voltage supply is measured for each probe by another A/D of the DS2438 [29] and applied in the calibration curves each time a humidity data is measured. Sensors are synchronized and their data recorded by an ATMega328 microcontroller (Microchip Technology Inc., Chandler, AZ, USA) as described in [11].

### 2.3. Sensors Location

**Figure 1:**
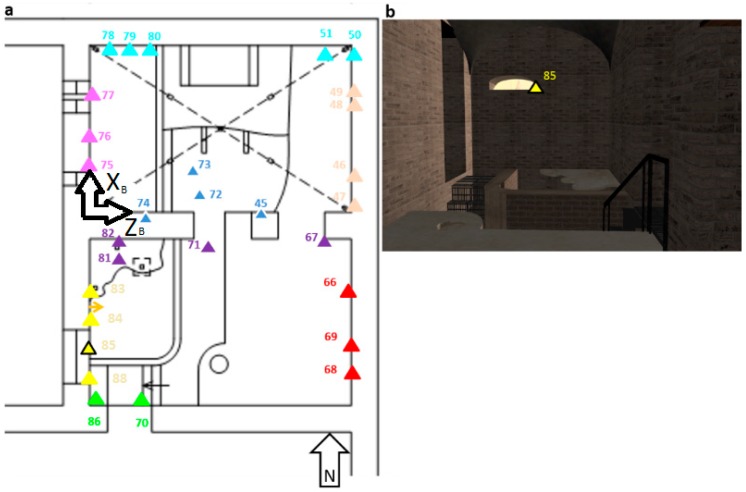
(**a**) Sensor locations (numbered triangles); (**b**) Sensor #85 (yellow triangle), outdoor climate control. Each sensor has a different color due to wall belonging. Each wall has a reference system (*xyz*) to individuate the sensor’s position (coordinate allocation is expressed in Table 1). Legend: pink color corresponds to wall A, fuchsia color corresponds to wall B, azure corresponds to wall C, blue corresponds to wall D, yellow corresponds to wall E, red corresponds to wall F, green corresponds to wall G and violet corresponds to wall H.

A total of 29 probes were installed, 28 in the interior of the *Mithraeum* and *pre-Mithraeum* of Casa di Diana (Figure 1a), and one additional probe placed on the sill of a window as an outdoor climate control (Figure 1b). The coordinate locations (*xyz*) for sensors along the walls are described in Table 1, as it has been drawn on wall B (Figure 1, fuchsia). The origin started at the left corner of the wall and at the pedestrian height. The coordinate *x* indicates the horizontal distance to the origin, coordinate *y* indicates the vertical distance to the origin, coordinate *z* indicates the distance to the walls surface only for two sensors (#72, #73) that are not placed along the wall. 

### 2.4. Database

The continuous monitoring campaign system was started on 29 June 2014 and finished on 21 May 2015. The records of March 2015 were completely missing. In order to avoid possible abnormal measurements, data after 31 January 2015 were disregarded for the analysis. 

The system records data with a frequency of 60 data points per hour (1 data per minute) being capable of recording 43.200 data points per month (30 days × 24 h/day × 60 data points/h). In this study we have a data base of 23,302,080 data, composing a data matrix of 401.760 rows (279 days × 24 h × 60 min) and 58 columns (29 sensors T + 29 sensors RH). Note that, sensors T#81-88 did not work during the month of August. On the other hand, sensor RH#77 was completely discarded due to instrument anomalies, during the entire data recording. Data were stored in Burrito software [31].

### 2.5. Data Analysis

The methodological procedure of the paper is as follows. First, an exploratory analysis of the thermo-hygrometric data is carried out to characterize the archaeological site. The starting hypothesis is coincident with [17], where events that contribute to upsetting the microclimatic balance considered the main source of damage. After, we select a subsample of sensors that present the ideal characteristics to model the hourly variation.

With this subsample, a multiple linear regression analysis and a logistic regression analysis are performed to assess the possible influence of the visitors in the upset of the microclimatic equilibrium.

Principal Component Analysis (PCA) was performed using Unscrambler version 9.7 (a chemometric software package from Camo, Woodbridge, NJ, USA), with a cross validation method. Other analyses were performed with the software packages OriginPro 8.5.1 (OriginLab, Northampton, MA, USA) and SPSS 22 (IBM, North Castle, NY, USA).

#### 2.5.1. Dimension Reduction Techniques: PCA and Correspondence Analysis

PCA is a dimension reduction technique. It is an exploratory method whose objective is to summarize a large amount of data in a small number of dimensions, with the least possible loss of information. PCA looks for the projection according to which the data are best represented in terms of least squares, converting a set of observations of possibly correlated variables in a set of variable values without linear correlation called principal components.

This linear transformation is constructed through the covariance matrix or matrix of correlation coefficients. Due to the symmetry of this matrix, there is a complete set of eigenvectors and eigenvalues that lead to the transformation of the old coordinates into the coordinates of the new base.

The Correspondence Analysis is a statistical technique that is used to analyze, from a graphical point of view, the dependency relations of a set of categorical variables from data of a contingency table. Its objective is similar to that of PCA, but applied on categorical variables. Inertial matrices for columns and rows are calculated and diagonalized, obtaining their eigenvalues and eigenvectors, defining a space formed by the dimensions and the projection of the categories on it.

#### 2.5.2. Multiple Linear Regression and Logistic Regression Models

The multiple linear regression considers that the values of the dependent variable (*Y*) have been generated by a linear combination of the values of *k* explanatory (independent) variables (*x*) and a random term (error or residual, *u*):

(1) $$Y=\beta_{0}+\beta_{1}x_{1}+\beta_{2}x_{2}+\ldots +\beta_{k}x_{k}+u$$

The coefficients are estimated minimizing the residual variance. Balance is understood as the stability of microclimatic conditions. The hourly variation of temperature represents the deviation from the balance conditions and, therefore, possible source of deterioration [17]. Thus, in our study, the hourly variation of temperature is selected as the variable of interest.

The independent variables considered in the linear regression analysis are the continuous variable *visitors* (taking values from 0 to 60), and the dichotomous variable *visit*, which takes the value 1 on the day and time that a guided visit takes place in Casa di Diana. In addition, the control variable considered in the model are the sensor variable, the dummy variables month (*July*, *September*, *October*, *November*, *December*, taking January as a reference), the difference between the outdoors temperature and the monthly moving average of the outdoors temperature (*EXT85_MA*) and the dichotomous variable *EXT85d* which is equal to 1 when the hourly variation of the outdoors sensor exceeds the mean hourly variation of the outdoors sensors for that month, considering a moving average.

According to the characterization of Casa di Diana, a subsample of sensors of temperature is selected, since it is intended to observe variations in the temperature that in principle are not derived from the architectural characteristics of the place (see Section 3.1 and Section 3.2). Finally, for the linear regression analysis a sample of 793,620 data, arranged in a matrix with 66,135 rows and 12 columns, is considered.

Note, that the models are replicated for the case in which the variable *visitors* are considered as quantitative variable. As worse adjustment results are obtained only the final model are presented in the section of results. The interaction of the variables *month* and *visit*, and the triple interaction of the variables *month, visit* and *cluster* are also considered. However, the adjustment of the models is not modified and, according to the parsimony principle, the simplest model is selected. On the other hand, logistic regression models the outcome of a categorical variable as a function of the independent variables. The logistic regression analysis is framed within the set of Generalized Linear Models (GLM) that uses the logit function as a link function. The model takes the following form:

(2) $$p=\frac{1}{1+e^{-\left(\beta_{0}+\beta_{1}x_{1}+\beta_{2}x_{2}+\ldots +\beta_{k}x_{k}+u\right)}}$$

The odds ratio is a measure of how much higher/lower is the probability that an event will happen than the probability that it will not occur. It is calculated as:

(3) $$\frac{p}{1-p}=e^{\beta_{0}+\beta_{1}x_{1}+\beta_{2}x_{2}+\ldots +\beta_{k}x_{k}+u}.$$

In Section 3.3, a logistic regression mathematical model is developed to determine the effect of visitors on the probability that the hourly variation represents a deviation from the equilibrium conditions; that is, when it presents a value higher than the monthly average value. The dummy variable *HV_largerMHV* (hourly variation larger than mean hourly variation) is defined. *HV_largerMHV* will take the value 1 when the hourly variation is larger than the monthly average of the hourly variation of the sensor, considering a monthly moving average, and the value 0 otherwise. It is understood that hourly variations larger than the monthly average hourly variation are likely to be considered upset of thermal balance.

## 3. Results and Discussion

### 3.1. Characterization of “Casa di Diana”

**Figure 2:**
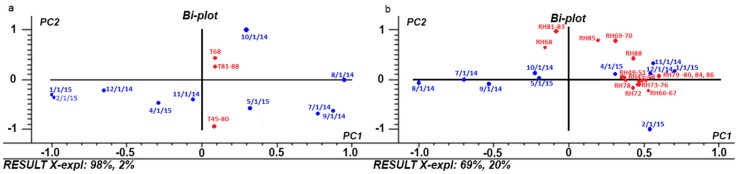
PCA Bi-plot (scores and loadings). (**a**) Tempera–ture PC1-PC2; (**b**) Relative Humidity PC1-PC2. Legend: blue color (scores), red color (loadings), *X-expl* refers to the percentage of variability of each sensor (*X*) explained by components 1 (PC1) and 2 (PC2), respectively.

A PCA is performed on the original data in order to characterize the archaeological site. The dataset was previously auto scaled and a model with two principal components (T), explaining the 100% of the total variance (Figure 2a), and two principal components (RH), explaining the 89% of the total variance (Figure 2b), is selected to fit the data.

Regarding temperature (Figure 2a), two clear groups are identified. One group is comprised by 8 sensors (#68, #81–#88) that were situated between the south west *pre-Mithraeum* wall and the north *pre-Mithraeum* wall (Figure 1a—yellow and orange triangles). This is the more ventilated area due to the window and main entrance. All the rest of the sensors (#45–#80) follow the same behavior. There is not found a particular relationship between wall and season (except for October, that seems to have an own trend). Regarding RH (Figure 2b), more data variability is found and two groups are identified. One group is composed by sensors with different behaviors and located in a more ventilated area. The other group comprises sensors located along the more protected walls, away from openings, windows and frontal walls, where the outdoor exchanges are frequent, and present very similar behavior between them. 

**Figure 3:**
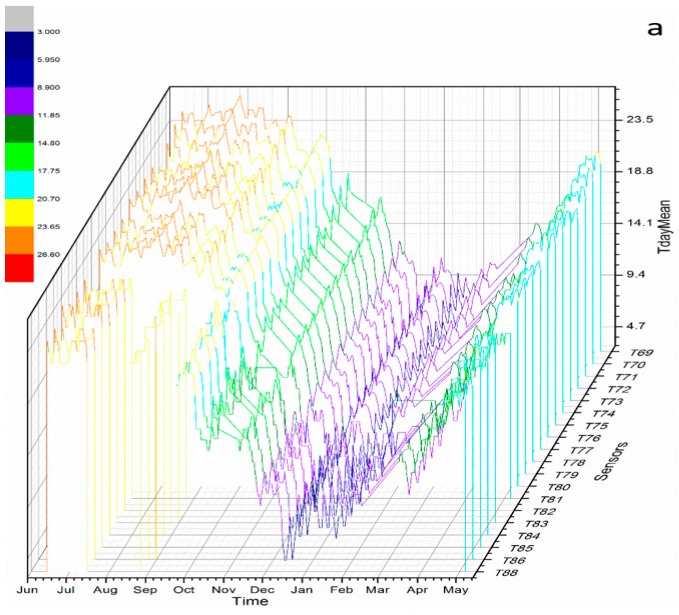

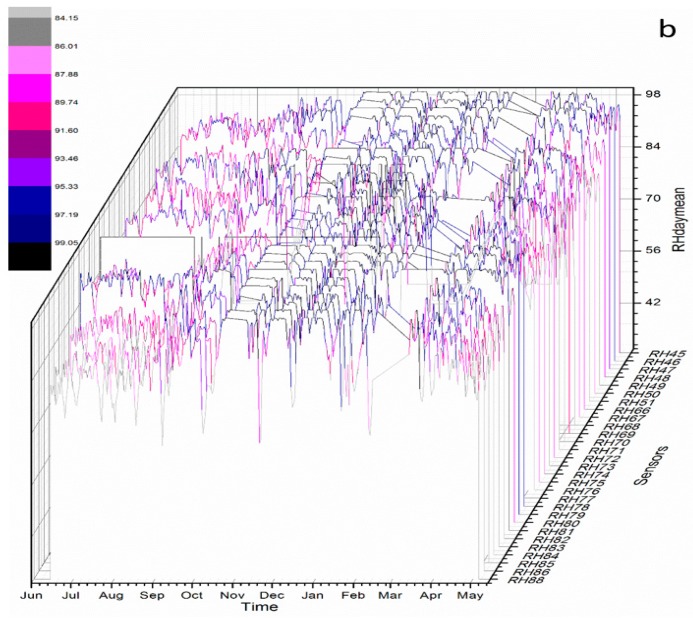
Multi-Curve Plot. (**a**) Tday_m_ vs. time; (**b**) RHday_m_ vs. time. Sensor locations are indicated in Figure 1.

In order to understand the thermo-hygrometric behavior during the year, Multi-Curve plots for the daily mean temperature (Tday_m_) and RH (RHday_m_) are represented for each sensor (Figure 3). In general, Tday_m_ remains stable between July and at the end of October (26.6–20.7 °C). The higher amplitude is present during the winter season (17.7–3 °C, Figure 3a). Regarding RHday_m_, it remains stable along the entire monitoring campaign with values between 99–84% (Figure 3b). Note that during the autumn-winter, the RH values are closed to saturation (97–99%) (Figure 3b). 

Taking into account the walls behavior, the sensors are grouped by wall belonging and the maximum and minimum daily values for T and RH are calculated (Tmax/Tmin and RHmax/RHmin, respectively). 

**Figure 4:**
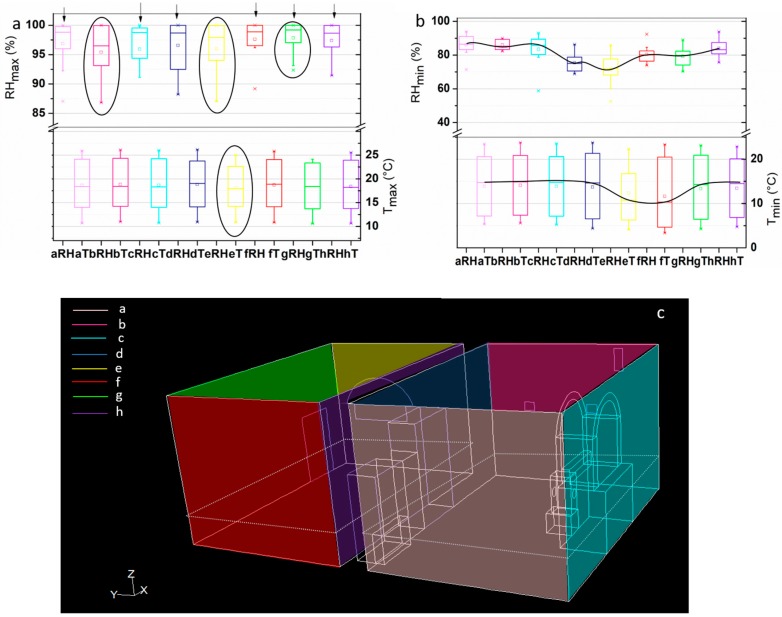
(**a**) Box & Whisker Tmax and RHmax; (**b**) Box & Whisker Tmin and RHmin; (**c**) 3D representation. The colors are according with the wall belonging.

Regarding Tmax (Figure 4a), it remains stable along the walls. Sensors in wall “e” seem to have less variability than sensors on the other walls. RHmax has more variability; sensors in walls “a, c, d, f, h” have less data variability than sensors in walls “b, e, g”. The difference between those two groups is in accordance with the presence or absence of air flow, as the first group (more data variability) was directly exposed to air flow (Figure 4c). 

The minimum values of T and RH corroborate the main results addressed by the maximum values. In addition to the previously considered factors, high levels of RH (rising damp, presence of probably seeping water) and wall temperature lower than air temperature induce surface condensation. Temperature sensors installed in previous monitoring campaigns and different materials indicated that the temperature of rock and mortars was always lower than air temperature, subsequently inducing water condensation on the surfaces of the material surfaces, as well as efflorescence and sub-efflorescence [15,16].

### 3.2. Modelling the Impact of Visitors on the Hourly Variation

According to the characterization, the data of the sensors #45–#80, excluding sensor #68, are taken, since it is intended to observe variations in the temperature that in principle are not derived from the architectural characteristics of the place. The HR data has been disregarded since it has been very constant during the whole monitoring campaign. Furthermore, the hours when there was no data for the outdoors sensor (#85) have been eliminated from the sample.

The hourly variation of temperature is selected as dependent variable. The independent variables considered in the analysis are variable visitors, visit, month (*July*, *September*, *October*, *November*, *December)*, *EXT85_MA* and the dichotomous variable *EXT85d*. 

A first stepwise regression analysis results in an adjusted *R*^2^ of 39.2%. The model is significant with *p*-value < 0.001 and the coefficients of the significant variables appear in Table 2. The visits increase the hourly variation, with an aggravating effect when the outdoors hourly variation is also higher than its expected monthly average. Note that when a visit takes place the hourly variation increases by 0.106 °C; when the hourly variation is also higher than its expected monthly average and a visit takes place, it results in an increase of 0.17 °C (0.106 + 0.064).

**Figure 5:**
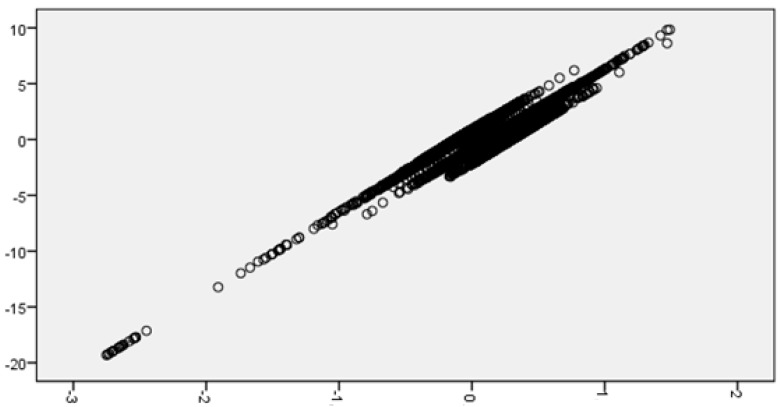
Scatter plot of the standardized residuals (vertical axis) according to the dependent variable (horizontal axis).

It can be deduced from the analysis of the residuals that there are four clearly identifiable data clusters with a different regression line (Figure 5). A cluster analysis of the residuals is performed and the conglomerates (hereinafter, clusters of residuals) have the characteristics specified in Table 3.

A priori it seems reasonable that these groups of observations with a particular relationship with the dependent variable may correspond to sets of sensors with common temperature characteristics (average and variability) caused by their placement in the archaeological site, despite the fact that the sample consists of the sensors with more similar behavior. However, a correspondence analysis for the variable cluster of residuals and the categorical variable sensor does not seem to reveal any direct relationship between the categories of both variables. In addition, to analyze this possibility, a regression analysis is performed, including as categorical variables the groups of sensors that seem to have a similar behavior according to the perceptual map of correspondence analysis. None of these variables is significant, so the influence of the sensor variable is discarded.

Therefore, it can be deduced that the clusters of residuals are related to specific observations (independent of the sensor) with the influence of variables not considered in the model, such as ventilation or solar radiation, for example.

The aim of the work is to evaluate the influence of visits on the upset of the thermal equilibrium of the archaeological site, so it was decided to carry out a second regression model including the clusters of residuals that allows evaluating the differences between these subgroups. Future research will be necessary to characterize these clusters so that prevention measures can be designed in the most appropriate way. A Computational Fluid Dynamics study is being developed. Air sensors have been installed and, in the future, the recorded data will allow improving the evaluation of the model residuals [32].

With the introduction of three dummy variables that allow modeling the four clusters of residuals, and all their interactions with the rest of the independent variables, a model with an adjusted *R*^2^ of 85.4% is obtained, which represents a great improvement in the predictive capacity with respect to the first model. The model is significant with *p*-value < 0.001 and the coefficients of the variables introduced in the final model appear in Table 4.

The hourly variation depends on the month as stated by the parameters of the variables *July*, *September*, *October*, *November*, *December*. As these parameters are negative, *January* is the month historically presenting the larger hourly variations in temperature. Depending on the cluster, the effect of the month is increased or reduced.

The visits always increase the hourly variation, with an aggravating effect when the outdoors hourly variation is also higher than its expected monthly average. Note that when a visit takes place the hourly variation increases by 0.031 °C for observations belonging to cluster 1; when the hourly variation is also higher than its expected monthly average and a visit takes place, it results in an increase of 0.118 °C for cluster 1. Furthermore, observations of clusters 3 and 4 present a greater sensitivity to visits. When a visit takes place, the hourly variation increases of 0.106 °C and 0.097 °C for observations belonging to cluster 3 and 4, respectively. This significant effect of visits on the thermal upset is in line with the findings of the study of CO_2_ [26,27]. 

**Figure 6:**
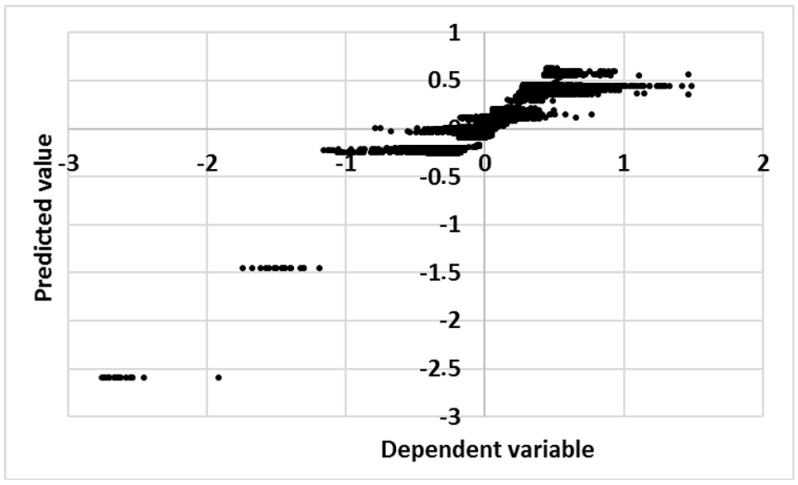
Predicted value versus dependent variable of the model.

**Figure 7:**
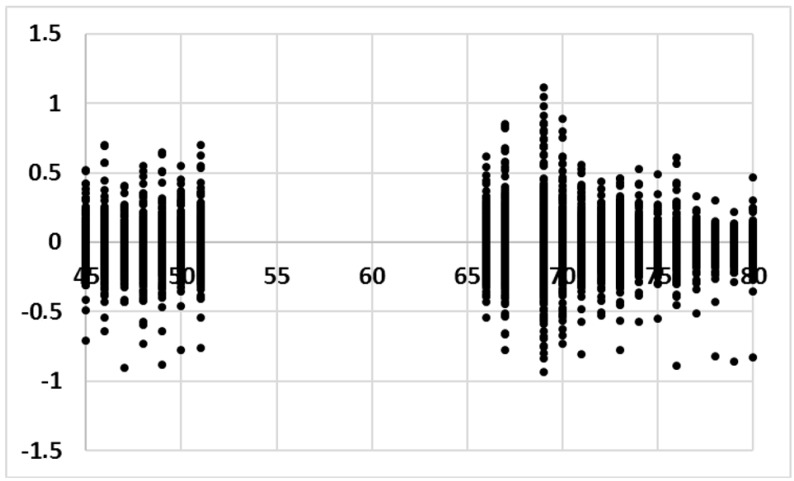
Residuals of the model (vertical axis) depending on the sensor (horizontal axis).

An analysis of the residuals of the model (Figure 6) shows that new clusters appear. Despite this, we have been able to identify the influence of visitors, which is the main objective of the paper. An individualized analysis per sensor has discarded the possibility of autocorrelation of residuals. In addition, abnormal residuals are present in all sensors, which surely must coincide with some of the identified clusters reinforcing the idea that these clusters are related to some external variables not considered in the model. Sensor #69 has the largest residuals variance (Figure 7).

### 3.3. Modelling the Impact of Visitors on the Upset of the Thermal Balance

In this section, a logistic regression mathematical model is developed to determine the effect of visitors on the probability that the hourly variation represents a deviation from the equilibrium conditions. *HV_largerMHV* is the dependent variable; the independent variables considered in the analysis are the dichotomous variable visit, *EXT85_MA* and the dichotomous variable *EXT85d*. 

The Hosmer Lemeshow test tests the null hypothesis that the fitted model is correct, therefore as *p*-value > 0.05 so there is lack of evidence against the null hypothesis. The model has a Cox and Snell’s *R*^2^ equal to 0.418 and a Nagelkerke’s *R*^2^ equal to 0.567. Variable *EXT85_MA* resulted non-significant.

The model takes the following form:

(4) $$odds=\frac{p}{1-p}=e^{-2.105+3.538EXT85d+2.635VISIT-0.569EXT85d\times VISIT}$$

Thus, when a visit takes place the probability that the hourly variation reaches values higher than its monthly average is 10.64 times the probability that the balance is maintained, when the rest of the variables remain constant.

The classification table (Table 5) shows the number of cases that have been classified (predicted) by the model as 0 or 1 and compares with the original classification (observed) for each case (observation). In the classification table (Table 5) we can verify that our model has a high specificity (87.4%) and a remarkable sensitivity (83.6%). T default limit point of the calculated *Y* probability is set to 50% (0.5). It has been tested that, despite decreasing the limit point, the classification is maintained.

Note that no improvement of the model is achieved when including the variable visitors as quantitative variable.

## 4. Conclusions

The innovative of the sensors technology—high memory of data storing (10 years and more) and high frequency of data recording (1 data/min)—allow us obtaining a big database and modelling, for the first time, the influence of visitors in the microclimate of an archaeological site. 

A prior exploratory analysis of the data allowed to individuate the more humid walls, the walls more protected to air flow, as well as individuating differences into the single building (between the *Mithraeum* and *pre-Mithraeum*). 

Exploratory analyzes have shown that Casa de Diana has a stable microclimate in terms of RH and that the largest variations occur in temperature and during autumn-winter. This paper is based on the idea that the main cause of damage in cultural heritage sites is the upset of the balance of microclimatic conditions. For the first time, the upset of equilibrium is mathematically modeled both in terms of hourly variations and in terms of the probability of significant changes.

The multiple linear regression model stated the influence of the visits on an increase in the hourly variation of the temperature. However, the residuals of the model determined the existence of observations with linearly differentiated behaviors. Therefore, more research is needed including other variables such as ventilation and solar radiation, with the purpose of identifying and characterizing the observations included in these clusters.

The logistic regression model assessed that when a visit takes place the probability of the upset of the thermal balance is 10.64 times larger than the probability that the balance is maintained.

Due to the high humidity of the archaeological site, the visitors did not influence this parameter; however, they affect the temperature despite having open windows to the outdoors and no mechanism of temperature control.

Note that the main goal of the study is proposing a monitoring system and a mathematical modelling methodology for the evaluation of the significant influence of visitors, more than the quantification of the damage, serving as a guide for other CH sites. In order to quantify the damages produced by the visitors, a second study without visitors should be carried out in the future.

## Figures and Tables

**Table 1:** Sensors description and coordinated allocation.

Reference System	Sensors N.	*x* (cm)	*y* (cm)	*z* (cm)
BLUE	45	70	102	0
	72	424	0	94
	73	400	80	116
	74	512	140	0
PINK	46	372	192	0
	47	372	89	0
	48	197	142	0
	49	196	304	0
FUCHSIA	75	126	163	0
	76	176	302	0
	77	294	93	0
AZURE	50	504	146	0
	51	425	201	0
	78	88	139	0
	79	174	148	0
	80	190	197	0
GREEN	70	226	217	0
	86	560	136	0
YELLOW	83	330	90	0
	84	245	120	0
	85	164	204	0
	88	32	169	0
RED	66	7	70	0
	68	242	154	0
	69	205	280	0
VIOLET	67	532	164	0
	71	290	106	0
	81	84	−12	0
	82	86	80	0

**Table 2:** Estimated coefficients of the independent variables (first multiple linear regression model).

Parameter	Coefficient	Significance	Parameter	Coefficient	Significance
*Constant*	−0.084	0.000	*June*	−0.042	0.000
*EXT85d*	0.217	0.000	*July*	−0.023	0.000
*EXT85_MA*	0.001	0.003	*September*	−0.006	0.003
*VISIT*	0.106	0.000	*December*	−0.017	0.000
*EXT85d* * *VISIT*	0.064	0.000			

**Table 3:** Characteristics and compositions of the clusters of residuals.

Cluster	Number of Cases	% Cases	Center
1	19,349	29.26	−0.13525
2	38	0.06	−2.00502
3	5,863	8.87	−0.2594
4	40,885	61.82	0.02867
Total	66,135	100.00	

**Table 4:** Estimated coefficients of the independent variables (second multiple linear regression model).

Parameter	Coefficient	Significance	Parameter	Coefficient	Significance
*Constant*	−0.214	0.000	*cl4*	0.161	0.000
*EXT85-Mont_av*	0.002	0.000	*cl3* * *VISIT*	0.075	0.000
*EXT85d*	0.217	0.000	*cl4* * *VISIT*	0.066	0.000
*VISIT*	0.031	0.000	*cl3* * *EXT85d*	0.031	0.000
*EXT85d* * *VISIT*	0.087	0.000	*cl2* * *November*	−1.155	0.000
*June*	−0.045	0.000	*cl3* * *June*	−0.088	0.000
*July*	−0.036	0.000	*cl3* * *July*	−0.044	0.000
*September*	−0.011	0.000	*cl3* * *September*	−0.068	0.000
*October*	−0.01	0.000	*cl3* * *October*	−0.036	0.000
*December*	−0.016	0.000	*cl3* * *December*	−0.069	0.000
*cl2*	−1.232	0.000	*cl4* * *July*	0.009	0.000
*cl3*	0.407	0.000	*cl4* * *September*	−0.005	0.027

**Table 5:** Classification table of the logistic regression model.

Observed			Predicted	
		0	1	Percentage
***HV_largerMHV***	**0**	35,078	5066	87.4
	**1**	4275	21,716	83.6
**total**		39,353	26,782	85.9
